# Reviving cancer vaccines in renal cell carcinoma: a new chapter in immunotherapy for kidney cancer

**DOI:** 10.1016/j.iotech.2026.101611

**Published:** 2026-09-07

**Authors:** F.M. Scallo, J.J. Ishizuka, D.A. Braun

**Affiliations:** 1 Center for Molecular and Cellular Oncology, Yale Cancer Center, Yale School of Medicine, New Haven, USA; 2 Section of Medical Oncology, Department of Internal Medicine, Yale School of Medicine, New Haven, USA; 3 Department of Pathology, Yale School of Medicine, New Haven, USA; 4 Department of Immunobiology, Yale School of Medicine, New Haven, USA; 5 Department of Urology, Yale School of Medicine, New Haven, USA

**Keywords:** renal cell carcinoma, cancer vaccines, neoantigens, tumor-associated antigens, immunotherapy, tumor microenvironment

## Abstract

Renal cell carcinoma (RCC) remains one of the most immunologically responsive solid tumors, yet durable benefit from immune checkpoint inhibitors (ICIs) is achieved in only a subset of patients. Although ICIs are conventionally thought to primarily act by reinvigoration and expanding preexisting antitumor T-cell responses, their ability to consistently broaden tumor-specific immunity remains limited, highlighting a potential role for therapeutic cancer vaccines in RCC to expand, activate, and diversify tumor-targeting T-cell populations. Vaccine strategies in RCC have evolved from broad autologous whole tumor antigen (WTA) approaches to defined tumor-associated antigen (TAA) platforms and, more recently, personalized neoantigen vaccines enabled by next-generation sequencing. Early WTA vaccines demonstrated feasibility and immunogenicity, particularly in the adjuvant setting, but failed to consistently improve survival in metastatic disease. TAA-directed vaccines targeting Wilms’ tumor 1, survivin, mucin 1, carbonic anhydrase IX, and 5T4 confirmed on-target immune activation but yielded limited clinical benefit. Personalized neoantigen vaccines, particularly in high-risk resected clear cell RCC, have shown robust and durable T-cell responses with encouraging early clinical signals and are being investigated in larger clinical trials. However, vaccine efficacy in RCC is constrained by tumor-intrinsic immune suppression, heterogeneous antigen presentation, and subtype-specific tumor immune microenvironments. Emerging strategies to further modulate immunosuppressive signals from tumor cells and the microenvironment may enhance vaccine responsiveness, particularly in immune-cold subtypes. Future progress will require stage-specific application, improved antigen selection, and rational combinatorial approaches tailored to RCC biology.

## Highlights

• Checkpoint inhibitors rarely produce durable antitumor immunity in RCC.
• WTA and TAA vaccines were safe and immunogenic but had limited benefit.
• Personalized neoantigen vaccines can induce durable T-cell responses.
• Key questions: optimal targets, clinical setting, and combination therapies?
• RCC vaccines should be subtype- and stage-specific with rational TME modulation.

## Renal cell carcinoma: A promising target for cancer immunotherapies

Globally, renal cancers including renal cell carcinoma (RCC) ranked as the sixth and ninth leading new cancer diagnosis for men and women, respectively, with 434 840 individual cases recorded in 2022.^1^^,^^2^ RCC subtypes include clear-cell RCC (ccRCC), which comprises 70%-80% of cases, and ‘nonclear’ or variant histologies, including papillary RCC (pRCC; 13%-20% of cases) and chromophobe RCC (chRCC; ∼5% of cases).^3^ Large-scale genomic efforts such as The Cancer Genome Atlas have aided in characterizing the molecular landscapes of these subtypes, laying the groundwork for targeted agents that would come to shape current therapeutic strategies.^4^

Beyond targeted therapies (including antiangiogenic tyrosine kinase inhibitors and small molecule inhibitors of HIF2α and mTOR), RCC has long been regarded as an immunogenic tumor, with spontaneous regression and durable remission to cytokine therapy including interleukin 2 (IL-2) and interferon-α (IFN-α) in a small subset of patients.^5^^,^^6^ These early clinical observations provided foundational evidence for preexisting tumor-specific T cell immunity within RCC tumors and foreshadowed the success of immune checkpoint inhibitors (ICIs), which now define the most recent therapeutic wave in treating metastatic RCC (mRCC).^5^, ^6^, ^7^, ^8^ The clinical activity of ICIs in RCC, particularly in ccRCC, is exemplified by the phase III CheckMate 214 trial^8^ and multiple other ICI-based trials,^9^, ^10^, ^11^ which demonstrated that releasing immunologic ‘brakes’ can reinvigorate preexisting T-cell populations and elicit clinically meaningful antitumor responses compared with the previous standard of care, sunitinib. However, durable benefit continues to remain elusive for most patients in the metastatic setting.^12^ Although ICIs enhance T cell function by relieving inhibitory signaling, they do not directly provide a source of tumor antigens necessary for initiating *de novo* T cell priming, nor do they inherently direct antigen-specific immune responses toward tumor cells. These challenges highlight a central issue in RCC immunotherapy: effective and durable tumor control likely requires not only the removal of inhibitory signals but also strategies that actively provide tumor-specific antigens and guide the priming of targeted T-cell responses.^13^

### Rationale for a cancer vaccine in RCC

Therapeutic cancer vaccination seeks to overcome this limitation by deliberately exposing the immune system to a diverse and clinically relevant source of tumor antigens, both enabling priming of new T-cell responses and potential expansion of previously primed, antigen-specific T cells.^14^ Within the framework of the cancer-immunity cycle, the generation of effective CD8 T cell-mediated immunity requires tumor cells undergoing apoptosis release antigens that are captured, processed, and presented by professional antigen-presenting cells (APCs).^15^ These APCs subsequently prime and activate naïve T cells in the lymph nodes, which then traffic to and infiltrate the tumor, recognize malignant cells, and mediate cytotoxic killing, thereby perpetuating the immunity cycle. Although tumor antigens capable of eliciting strong immunogenicity have been extensively characterized in other malignancies, most notably in melanoma, the antigenic landscape of RCC remains comparatively underdefined.^16^^,^^17^ In this review, we discuss the evolution of antigen discovery underlying cancer vaccine strategies in RCC, spanning early whole tumor antigen (WTA)-based approaches, subsequent efforts targeting defined tumor-associated antigens (TAA), and more recent personalized neoantigen vaccines. Finally, we will address the key biological and clinical challenges that have limited vaccine efficacy in RCC, along with emerging opportunities and future directions to overcome these barriers.

## Vaccine approaches in RCC: Past and present

### Autologous tumor vaccines

Early vaccine studies in RCC utilized WTA, prepared from autologous tumors obtained at nephrectomy, processed into a single-cell suspension, devitalized to ensure safety, and readministered on defined dosing schedules.^18^ This process preserves the tumor’s full antigenic repertoire including neoantigens, TAAs, and when present, viral antigens such as human endogenous retroviruses (hERVs), allowing WTA vaccines to deliver a broad set of epitopes to professional APCs and thereby engage both CD8+ cytotoxic and CD4+ helper T cells to promote polyclonal antitumor responses.^19^ The downside of this approach is the potential to vaccinate with a large number of irrelevant or even tolerogenic antigens, dampening immune response and requiring reliance on exogenous adjuvants or tumor cell modifications to achieve effective antigen presentation and robust T cell priming.^20^ Select trials investigating the use of WTA vaccines in RCC are summarized in Table 1.

**Table 1:** Summary of selected WTA vaccine clinical trials in RCC

Vaccine trial	Method	Patient population	Combination/formulation	Phase (# of patients)	Key immunologic findings	Key clinical outcomes	Trial ID	Ref.
Galligioni et al^25^	Irradiated autologous tumor cells	Adj	BCG	Phase III (120)	DTCH responses to autologous tumor in 38/54 vaccinated patients; however, DTCH reactivity does not correlate with OS	No DFS/OS benefit: 5-year DFS 63% versus 72% (control); 5-year OS 69% vs 78%	—	^21^
Reniale	IFNγ-primed autologous tumor cells followed by repeated rapid freeze-thaw	Adj	—	Phase III (379)	No test for monitoring immune responses	Improved PFS: 5-year and 70-month follow-up were 77.4% and 72%, respectively, in the vaccine group and 67.8% and 59.3%, respectively, in the control group	—	^22^
Vitespen(HSPPC-96)	Purified autologous gp96–peptide complexes	Adj	—	Phase III (728)	Possible improvement in RFS in patients with early-stage disease who received Vitespen	No improved RFS; median follow-up of 1.9 years in Vitespen group was 37.7% and 39.8% in observation	NCT00033904	^23^
ADAPT(Rocapuldencel-T)	Autologous tumor RNA-loaded moDCs + CD40L RNA	1 L mRCC	Sunitinib	Phase III (462)	Immune responses detected in 70% of patients treated with vaccine; however, there exists an immunosuppressive TME barrier	No improved OS; median OS in combination group was 27.7 months and 32.4 months in SOC group	NCT01582672	^24^

Adj, postnephrectomy localized RCC; BCG, bacillus Calmette–Guérin; DFS, disease-free survival; DTCH, delayed-type cutaneous hypersensitivity; HSPPC-96, heat-shock protein peptide complex-96; IFNγ, interferon-gamma; moDC, monocyte-derived dendritic cell; mRCC, metastatic renal cell carcinoma; OS, overall survival; PFS, progression-free survival; RCC, renal cell carcinoma; RFS, recurrence-free survival; SOC, standard of care; TME, tumor microenvironment; 1 L, first-line; WTA, whole tumor antigen.

#### Early clinical experiences with WTA vaccines

Perhaps one of the first WTA vaccine efforts in RCC consisted of a phase I trial that investigated irradiated autologous tumor cells combined with the bacterial adjuvant *Corynebacterium parvum* in patients with metastatic disease. Four of 14 patients treated achieved objective responses in metastatic lesions, including one patient with regression of all 22 measurable metastases.^25^ Although limited by small size and lack of a control arm, these findings demonstrated the feasibility of broad autologous WTA vaccination and its ability to elicit clinically meaningful antitumor immunity in metastatic and adjuvant settings.

These findings, among other,^26^^,^^27^ helped support a larger investigation of a WTA vaccine with a randomized trial including 120 patients who underwent radical nephrectomy with half receiving three adjuvant intradermal vaccination with irradiated autologous tumor cells combined with adjuvant *bacillus Calmette–Guérin* (BCG).^21^ Most vaccinated patients developed delayed-type cutaneous hypersensitivity (DTCH) responses, reflecting successful tumor-specific T cell priming. Unfortunately, vaccination did not improve outcomes, with numerically lower 5-year disease-free survival (DFS; 63% versus 72%) and overall survival (OS; 69% versus 78%) compared with surgery alone. Further, the degree of DTCH response did not correlate with survival, highlighting the concept that simply assessing presence or absence of a vaccine response is an imperfect surrogate for clinical efficacy.

#### Reniale: IFNγ primed autologous whole-cell vaccine

The Reniale program introduced IFNγ priming of autologous tumor cells before devitalization to upregulate human leukocyte antigen (HLA)/antigen-processing machinery (APM) and adhesion molecules (e.g. intercellular adhesion molecules [ICAMs]) with the goal of maximizing tumor antigen presentation on the tumor cells before intradermal dosing.^28^^,^^29^ In a randomized, adjuvant phase III study, a total of 379 patients underwent surgical resection with 177 patients receiving vaccination. Reniale significantly reduced the risk of disease progression, with 5-year and 70-month progression-free survival rates of 77.4% and 72% in the vaccine group, compared with 67.8% and 59.3% in the control group.^22^ Vaccine was well tolerated with only 12 adverse events associated with treatment, reenforcing safety and proof of concept for adjuvant WTA vaccination as a new therapeutic for RCC in the adjuvant setting. Although encouraging, the European Medicines Agency did not grant approval for a number of reasons, including potential concerns around study design and methodology.^30^^,^^31^ Additionally, without a primary immunological readout of vaccine efficacy, it is difficult to dissect the role vaccine is playing in the improved therapeutic benefit observed.

#### Vitespen heat-shock protein gp96–peptide complex: gp96-peptide complex vaccine

A different autologous tumor-based approach involved purification of the heat-shock protein gp96–peptide complex (HSPPC-96) from patient tumors, based on the rationale that these complexes carry tumor-specific antigenic payloads and engage APCs via CD91, eliciting cross-presentation of peptide cargo on major histocompatibility complex class I (MHC-I).^32^ Autologous tumor-derived HSPPC-96 (Vitespen) was evaluated in a multicenter, open-label, randomized phase III trial (NCT0033904) in the adjuvant setting for high-risk RCC following nephrectomy. Patients were randomized to receive Vitespen (*n* = 361) or observation alone (*n* = 367), with recurrence-free survival (RFS) as the primary endpoint. At a median follow-up of 1.9 years, recurrence occurred in 37.3% of vaccinated patients and 39.8% of controls, demonstrating no significant improvement in RFS.^33^ Notably, a prespecified exploratory analysis revealed a trend toward reduced recurrence in patients with stage I-II disease treated with Vitespen (hazard ratio 0.576; *P* = 0.056), consistent with findings in earlier-stage melanoma,^33^ suggesting that WTA vaccine strategies may perform better in earlier-stage disease.

#### Rocapuldencel-T (AGS-003): tumor RNA-loaded dendritic cell vaccine

Building on the evolution from WTA to purified HSPPC-96, the field advanced to mature monocyte-derived dendritic cell (moDC) vaccines coelectroporated with amplified autologous tumor RNA and synthetic CD40L RNA, termed Rocapuldencel-T. These engineered moDCs present tumor antigens to CD8^+^ and CD4^+^ T cells via MHC class I and II while providing enhanced costimulatory signaling through CD40L. In a phase II trial (NCT0067819), Rocapuldencel-T was combined with the first-line tyrosine kinase inhibitor sunitinib, selected in part for its immunomodulatory effects, including suppression of myeloid-derived suppressor cells and regulatory T cells (Tregs).^34^^,^^35^ This combination induced antigen-specific T-cell responses and demonstrated encouraging survival signals, supporting the global ADAPT phase III trial treating newly diagnosed patients with metastatic ccRCC.^36^ However, the trial was halted for futility at interim analysis, with no OS benefit observed.^24^ Similar to the Vitespen trial, these results underscore the difficulty of achieving clinical benefit in the bulky metastatic setting, particularly without additional interventions to address the immunosuppressive tumor microenvironment (TME).

### TAA vaccines

A major limitation of WTA vaccines in mRCC is their reliance on surgical resection to obtain autologous tumor tissue, which is often impractical in patients with unresectable or metastatic disease. These challenges prompted the development of vaccines targeting defined TAAs. Ideal TAAs are minimally expressed in normal tissues, highly immunogenic, consistently expressed across tumors, and accessible to immune recognition. A major challenge for TAA vaccines is overcoming host tolerance, as T cell clones recognizing these self-like antigens are often deleted during thymic selection. In RCC, candidate TAAs include Wilms’ tumor 1 (WT1), survivin, mucin 1 (MUC1), carbonic anhydrase IX (CAIX), and 5T4, all of which have been evaluated in early clinical trials (Table 2).

**Table 2:** Summary of selected TAA vaccine clinical trials in RCC

Vaccine trial	Method	Patient population	Combination/formulation	Phase (# of patients)	Key immunologic findings	Key clinical outcomes	Trial ID	Ref.
WT1	WT1 peptide-pulsed autologous DC vaccine	mRCC	OK-432(TLR-4 ligand)	Phase I/II (5)	Induced measurable WT1-specific immune responses; decrease in Treg levels	Well tolerated; durable stable disease in subset; no objective responses	UMIN 000027279	^37^
Survivin/Telomerase	Survivin + telomerase peptide-pulsed autologous DC vaccine	mRCC	Low-dose IL-2	Phase I/II (27)	Antigen-specific T cell responses detected in evaluated patients (6/6 tested); induction of surviving-reactive CD8+ T cells	Well tolerate; stable disease >8 weeks in 13/27 patients; no objective responses	NCT 00197860	^38^
VEGFR1	HLA-A∗24:02 and HLA-A∗02 restricted VEGFR1 epitope peptide vaccine	mRCC	Montanide ISA-51VG incomplete	Phase I (18)	15/18 patients treated revealed positive CTL resonses	Well tolerated; 2 PR, 8 SD for 5-months	UMIN 000004911	^39^
TG4010(MUC1)	MVA-MUC1-IL2 viral vector vaccine	mRCC	IL-2 + IFNα	Phase II (37)	Induced/boosted MUC1-specific CD4+ and CD8+ T cell responses	Well tolerated; no improvement in objective clinical responses	—	^40^
CAIX	HLA-A∗24:02-restricted CAIX peptide vaccine	mRCC	—	Phase I (23)	CAIX-specific CTLs induced in 16/23 patients; humoral responses detected in all patients	Well tolerated; 3 PR, 6 SD,14 PD;	—	^41^
TRIST(TroVax)	Targeting 5T4	1 L mRCC	Sunitinib, IL-2, or IFNα	Phase III (732)	Cellular and humoral anti-5T4 responses in subset of patients	No improved OS; median OS was 20.1 months in the vaccine group and 19.2 months in the control group	NCT 00397345	^42^
IMPRINT(IMA901)	Multi-epitope HLA-A∗02-restricted TAA peptides (TUMAPs)	1 L mRCC	Cyclophosphamide pretreatment + sunitinib	Phase III (339)	Phase II: polyclonal CD8+ responses linked to OS; Phase III: diminished immune magnitude	No improved OS; median OS was 33.2 months in the combination group and not reached in the sunitinib-alone group	NCT 01265901	^66^

CAIX, carbonic anhydrase IX; CTL, cytotoxic T lymphocyte; DC, dendritic cell; HLA, human leukocyte antigen; IFNα, interferon-alpha; IL-2, interleukin-2; mRCC, metastatic renal cell carcinoma; MUC1, mucin 1; MVA, modified vaccinia Ankara; OS, overall survival; PR, partial response; SD, stable disease; PD, progressive disease; RCC, renal cell carcinoma; TAA, tumor-associated antigen; TLR, toll like receptor; TUMAP, tumor-associated peptide; VEGFR1, vascular endothelial growth factor receptor 1; WT1, Wilms tumor 1; 1L, first-line.

#### WT1

WT1 is a zinc-finger transcription factor that regulates growth factors and their receptors and is highly expressed across various tumors.^43^ WT1 peptide-based immunotherapy has shown activity in phase I/II trials including leukemia^44^^,^^45^ and solid tumors including lung^44^ and uterine.^46^ In advanced RCC, WT1 peptide-pulsed dendritic cell vaccination has been evaluated in HLA-A∗02 and HLA-A24:02-restricted patients. Clinically, four of five patients achieved stable disease, although no partial or complete responses were observed. Vaccination induced WT1-specific CD8^+^ T cell responses detected by HLA tetramer staining, with increased IFN-γ production, demonstrating antigen-specific immunity.^37^ Although WT1 remains a potential vaccine target, its variable and predominantly ccRCC-restricted expression (12.5%, 7/56 cases) highlights the need for more comprehensive analyses across RCC subtypes to better define its true prevalence.^47^

#### Survivin

Survivin is an inhibitor-of-apoptosis family protein with low/absent expression in most differentiated adult tissues, but is frequently upregulated in cancers, making it an attractive TAA for therapeutic vaccination.^48^ Survivin peptide-based vaccines have shown feasibility and immunogenicity in early-phase studies across solid tumors, including survivin-2B peptide trials in urothelial cancer^49^ and other malignancies.^50^^,^^51^ In mRCC, Berntsen et al^38^ evaluated an autologous dendritic cell vaccine loaded with a survivin plus telomerase peptide cocktail in HLA-A∗02 patients with concomitant low-dose IL-2. No objective responses were observed, but 13/27 patients achieved stable disease for >8 weeks, and antigen-specific immune responses were detected in all assessable patients tested (6/6), supporting on-target immunogenicity. Consistent with its candidacy as a shared target, survivin expression is significantly upregulated across ccRCC, pRCC and chRCC compared with normal kidney tissue, most prominently in ccRCC, with higher expression associated with worse OS in ccRCC and pRCC, further supporting its relevance as a therapeutic vaccine target.^52^

#### Vascular endothelial growth factor receptor 1

Vascular endothelial growth factor receptor 1 (VEGFR1) is a receptor tyrosine kinase that plays a central role in regulating angiogenesis and is upregulated in response to hypoxia in many solid tumors. In a nonrandomized, open-label phase I clinical trial, an HLA∗A02- or HLA∗A24:02-restricted VEGFR1 peptide was administered to 18 patients with mRCC, demonstrating a favorable safety profile with no toxicities grade 3 or greater. Antigen-specific cellular immunity was detected in 15 of 18 patients by IFN-γ ELISPOT, although clinical responses were limited, with two partial responses and eight cases of stable disease lasting >5 months.^39^ In a systematic review of 20 trials across several cancers, VEGFR-targeted peptide vaccines including VEGFR-1, VEGFR-2, combined VEGFR-1/VEGFR-2, and multi-epitope formulations were reported to induce antigen-specific immunologic responses in the majority of vaccinated patients, along with some evidence of clinical benefit (2.4% complete response, 26% partial response, and 44.4% stable disease).^53^

#### MUC1

MUC1 is a transmembrane glycoprotein expressed widely on the apical membrane of many glandular epithelia including in normal distal convoluted tubules and collecting ducts.^54^ In RCC, a systematic immunohistochemistry (IHC) study of 188 cases showed MUC1 protein expression with high prevalence across subtypes—84% in ccRCC, 65% in pRCC, and 91% in chRCC—supporting its candidacy as a shared target.^55^ In a phase II trial in mRCC, TG4010 (MVA-MUC1-IL2), continued in combination with IL-2 and IFNα, demonstrated feasibility and induced/boosted MUC-1-specific CD4+ and CD8+ T-cell responses. Despite being well tolerated and generating favorable adaptive responses, there was no improvement in clinical responses.^40^ To date, at least 10 clinical trials have been designed to target MUC1 across a variety of cancer types, highlighting its potential as a target for immunotherapies.^56^

#### CAIX/CA9

CAIX is a hypoxia-inducible, HIF-regulated transmembrane enzyme with high, diffuse membranous expression in ccRCC specifically.^57^ Early T cell-focused clinical efforts in mRCC tested CAIX-derived peptides. In a phase I trial restricted to HLA-A∗24:01-positive patients, vaccination was well tolerated and elicited peptide-specific cytotoxic T lymphocyte (CTL) responses in 16 of 23 patients and yielded clinical responses in nine patients, including three partial responses and six cases of stable disease.^41^ In a parallel approach, autologous dendritic cells transduced with a fusion-gene construct (granulocyte-macrophage colony-stimulating factor + CAIX) were shown to be safe and immunogenic in patients with mRCC, eliciting CAIX-specific immune responses and achieving stable disease in five of nine patients at 3 months, including one patient with durable disease stabilization lasting 27 months.^58^ Together, these early studies established biologic proof-of-concept for CAIX-targeted vaccination while highlighting the need for optimized adjuvants, patient selection, and combination in advanced disease.

#### 5T4/trophoblast glycoprotein

The oncofetal antigen 5T4 is normally restricted to placental tissue but is aberrantly expressed in multiple carcinomas, including RCC subtypes. Baseline humoral and T cell reactivity against 5T4 has been detected in a subset of patients with RCC, suggesting preexisting antitumor immunity.^59^ A modified vaccinia Ankara vector encoding 5T4 (MVA-5T4) was developed and evaluated in mRCC, in which early single-arm phase II trials demonstrated feasibility and immunogenicity, with most patients mounting *de novo* or boosted 5T4-specific cellular and antibody responses.^59^, ^60^, ^61^, ^62^

The TroVax Renal Immunotherapy Survival Trial evaluated whether MVA-5T4 could improve OS when added to first-line standard-of-care therapy in mRCC. In this randomized 1 : 1 trial, patients received up to 13 vaccinations in combination with either sunitinib, IL2, or IFN-α. Despite good tolerability, no survival benefit was observed (median OS 20.1 months with MVA-5T4 versus 19.2 months with placebo; *P* = 0.55).^42^ Notably, only 56% of patients developed detectable 5T4-specific antibodies, compared with over 80% in earlier-phase studies, implying reduced antigen-specific T cell priming.^59^, ^60^, ^61^, ^62^ Subsequent large-scale IHC analyses revealed lower and more heterogeneous 5T4 expression (∼40%-60% in ccRCC versus >95% initially), aligning with the modest humoral response rate observed in phase III, although exploratory analyses associated vaccine-induced 5T4 immunity with improved clinical outcomes.^63^^,^^64^

#### IMA901: a multi-epitope targeting TAA vaccine

Although earlier vaccine strategies targeted single TAAs, IMA901 incorporated multiple HLA-A∗02-restricted tumor-associated peptides (TUMAPs), including nine class I and one class II epitope. Although enrollment was limited to HLA-A∗02*-*positive patients, this represented one of the first multi-epitope TAA vaccines tested in RCC. In early-phase studies in mRCC, vaccination induced TUMAP-specific T cell responses, and patients responding to multiple epitopes were more likely to achieve disease control and improved OS compared with those with limited or absent responses.^65^ In these studies, cyclophosphamide pretreatment reduced Tregs by ∼20% and was associated with improved OS in immune responders, supporting immunomodulatory priming. Similar to ADAPT, sunitinib was hypothesized to further augment vaccine-induced T-cell responses by reducing immunosuppressive cell populations.^35^ These findings provided the rationale for combining IMA901 with cyclophosphamide and sunitinib in the phase III IMPRINT trial; however, no OS benefit was observed, with exploratory immune monitoring showing a significantly diminished magnitude of CD8-positive T cell compared with phase II.^66^ It is possible that concurrent sunitinib treatment blunted vaccine priming by impairing APCs.^67^^,^^68^ Despite negative phase III results, IMPRINT demonstrated that multi-epitope vaccination in RCC is feasible and immunogenic, but emphasized the need to overcome tumor immunosuppression and the limitations of vaccinating bulky metastatic disease alone.

### Neoantigen vaccines

Neoantigens are derived from tumor-specific mutations (e.g. somatic single-nucleotide variants and small insertions/deletions) that alter protein-coding sequences and create unique peptides not subjected to central tolerance. They are compelling targets for T cell-mediated antitumor immunity, with the aim of improving on-target efficacy while minimizing off-target toxicity.^69^^,^^70^ Although earlier WTA and tumor RNA-loaded DC vaccines likely contained neoantigens, advances in sequencing and computational prediction enabled truly personalized neoantigen vaccines. Although early neoantigen-targeting efforts focused on high-mutational burden tumors such as melanoma, recent trials in classically low-mutational burden tumors such as pancreatic and glioblastoma demonstrated that this approach is feasible more broadly.^71^^,^^72^ More recently, multi-epitope neoantigen vaccination in RCC has been shown to be feasible,^73^ building on earlier efforts targeting von Hippel-Lindau (VHL)-derived mutant neoantigens, the most common driver alteration in ccRCC, with selected neoantigen vaccine trials summarized in Table 3.

**Table 3:** Summary of selected neoantigen vaccine clinical trials in RCC

Vaccine trial	Method	Patient population	Combination/formulation	Phase(# of patients)	Key immunologicfindings	Key clinical outcomes	Trial ID	Ref.
VHL	Patient-specific VHL-derived peptides	mRCC	—	Phase I (5)	Peptide-reactive CD8+ T cell responses in 4/5 evaluated patients	No objective radiographic responses	—	^74^
PCV	Multi-epitope, patient-specific neoantigens	Adj	± Ipilimumab	Phase I (9)	All patients mounted vaccine-specific T cell responses; durable responses persisting months-years; antitumor reactivity in 7/9 patients via autologous tumor coculture	No RCC recurrences at median 40.2-month follow-up (high-risk, fully resected ccRCC)	NCT 02950766	^73^
INTerpath-004	Individualized neoantigen vaccine	Adj	Pembrolizumab	Phase II (ongoing)	—	—	NCT 06307431	

Adj, adjuvant; ccRCC, clear cell renal cell carcinoma; mRCC, metastatic renal cell carcinoma; OS, overall survival; RFS, recurrence-free survival; PCV, personalized cancer vaccine; HLA, human leukocyte antigen; RCC, renal cell carcinoma; VHL, von Hippel-Lindau.

#### Mutant VHL vaccine

VHL is one of the most common driver mutations in ccRCC, present in an estimated ∼60%-80% of cases.^75^ An early pilot study tested whether patient-specific VHL neoantigens—computationally predicted for high binding affinity to each patient’s class I HLA molecules—could be used as vaccine targets in metastatic ccRCC. Among six patients, two carried frameshift mutations that created entirely new 12-13-mer sequences, whereas the remaining cases had somatic single-nucleotide variants for which mutant eight-mer peptides were synthesized. Vaccine-induced cellular immunity was observed, with peptide-reactive CD8+ T cell responses in four of five assessable patients, but no objective radiographic responses were seen.^74^ Although no further development followed, this was the first trial to implement mutation-defined neoantigens as a precise, personalized vaccination approach in RCC.

#### Personalized cancer vaccines targeting multiple neoantigens

Although prior vaccination attempts provided a foundation, they were limited in either the approach or the clinical setting. Based on prior studies, a strategy that targets multiple epitopes (to avoid immune escapes), with neoantigens as the target (to avoid the need to overcome central tolerance), in a minimal disease setting (e.g. adjuvant) was developed. In a phase I trial, a personalized cancer vaccine (PCV) targeting multiple patient-specific neoantigens, given with or without ipilimumab, was tested in high-risk, fully resected ccRCC to assess immunogenicity and recurrence.^73^ All patients mounted vaccine-specific T-cell responses, with many of these being against the canonical RCC drivers such as mutations in *VHL*, *PBRM1*, *BAP1*, *KDM5C*, or *PIK3CA*. T-cell responses were durable, with vaccine-reactive T cells often persisting months or even years after the vaccination series was complete. Further, through coculture assays between vaccine-reactive T cells and autologous tumor cells, investigators demonstrated the induction of antitumor reactivity in seven of nine patients. Although only nine patients were treated in the initial study, limiting any interpretation of clinical results, it was notable that at a median 40.2-month follow-up there were no RCC recurrences. Evidence of both immunogenicity and absence of recurrence supports the idea that PCVs may be effective adjuvant therapy in fully resected RCC. A phase II randomized adjuvant trial evaluating an individualized neoantigen vaccine with pembrolizumab is ongoing (INTerpath-004; NCT06307431). These results are especially encouraging as neoantigen-targeting vaccines are showing signals of clinical activity in ICI-responsive cancers like melanoma (KEYNOTE-942; NCT03897881).^76^

## Building better RCC vaccines

### Optimizing vaccine strategies in RCC: patient- and tumor-specific considerations

**Figure 1:**
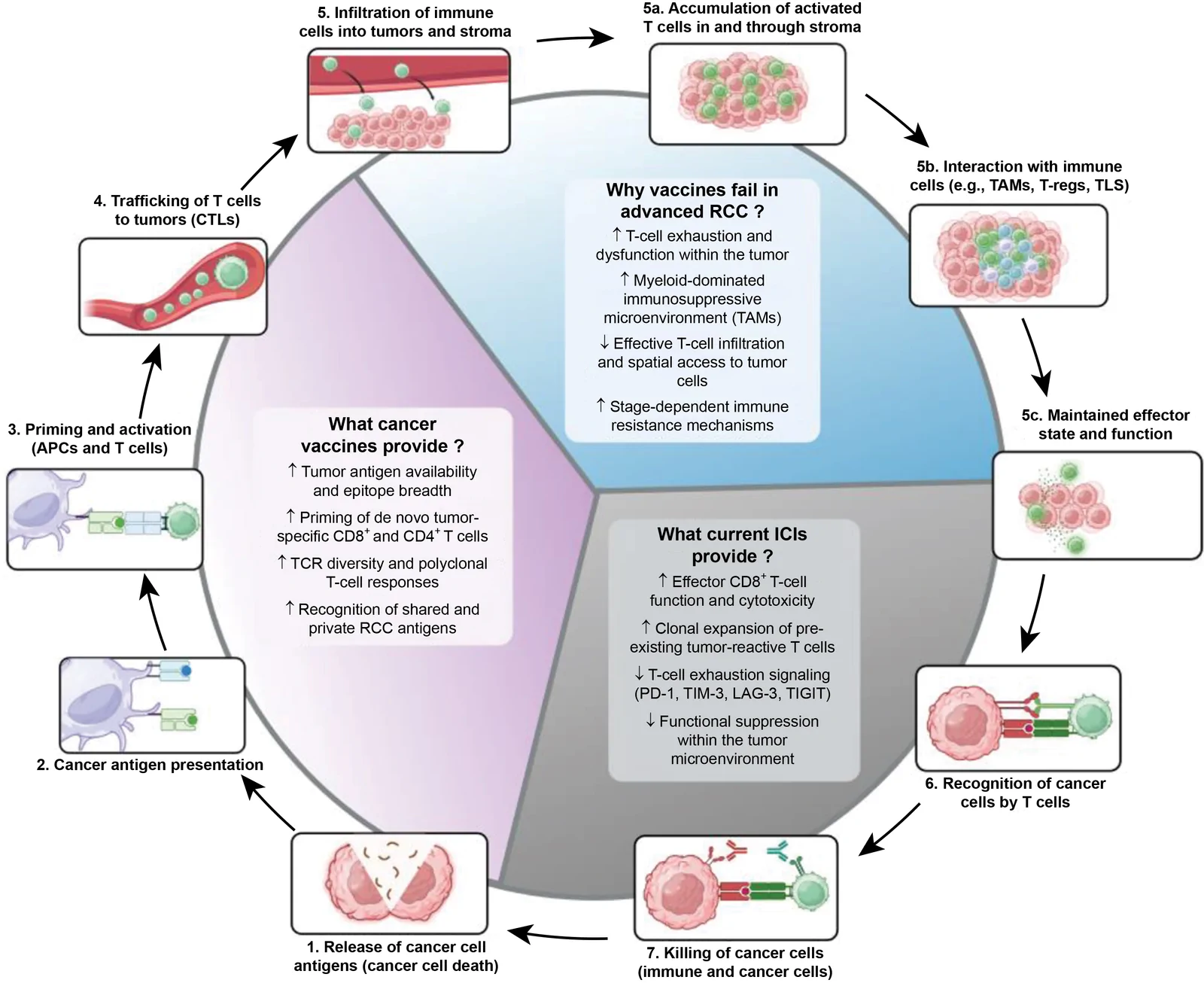
**Barriers to effective vaccination in RCC within the cancer immunity cycle.** Adapted from the cancer-immunity cycle framework proposed by Chen et al,^16^ this schematic illustrates the key steps required for effective antitumor immunity and how cancer vaccines and ICIs act at different stages. Vaccines promote antigen availability and priming of tumor-specific CD4^+^ and CD8^+^ T cells, expanding the TCR repertoire and enabling recognition of shared and private tumor antigens. In advanced RCC, vaccine efficacy can be limited by T cell exhaustion, immunosuppressive myeloid populations (e.g. TAMs), impaired T cell infiltration, and tumor-intrinsic resistance mechanisms. ICIs partially restore effector T cell function and clonal expansion but may remain insufficient without effective antigen priming and relief of tumor microenvironment-mediated suppression. APCs, antigen-presenting cells; CTLs, cytotoxic T lymphocytes; ICIs, immune checkpoint inhibitors; PD-1, Programmed cell death protein 1; RCC, Renal cell carcinoma; TAMs, Tumor-associated macrophages; TCR, T cell receptor; TLS, tertiary lymphoid structures.

Cancer vaccine design is inherently complex, encompassing factors such as delivery platform, adjuvant selection, and epitope composition that collectively shape the magnitude and quality of the immune response. Technical aspects of vaccine design and manufacturing have been comprehensively reviewed elsewhere,^77^^,^^78^ and consequently, this review will focus on antigen selection and T-cell responses within the framework of the cancer-immunity cycle in RCC specifically.^15^ In this context, effective cancer vaccination in RCC requires coordinated antigen priming, overcoming of tumor-intrinsic immune suppression, and reinforcement of effector T cell function (Figure 1, adapted from Chen et al^15^). These processes diverge across RCC subtypes, particularly ccRCC and chRCC, necessitating RCC subtype-specific vaccine design to achieve durable antitumor immunity.

#### Antigen priming and vaccine targeting in RCC

Although the antigen targets in ccRCC were more recently reviewed, the broader antigen landscape of RCC remains incompletely characterized, particularly in pRCC and chRCC.^17^ Neoantigens derived from recurrent driver mutations such as *VHL* and *PBRM1* represent compelling vaccine targets in ccRCC, a concept supported by the immunogenicity and clinical signals observed in the recent neoantigen vaccine trial.^73^ Further, clonal neoantigens (those that derive from mutations present in all cancer cells) have been associated with exceptional immune responses, highlighting their role as potential vaccine targets.^79^ Both ccRCC and pRCC exhibit comparable (modest) tumor mutational burdens (TMB), suggesting that similar neoantigen-based strategies may be feasible in pRCC (and consequently, patients with pRCC were eligible for the INTerpath-004 trial).^71^ In contrast, chRCC is characterized by a markedly lower TMB, limiting the availability of mutation-derived neoantigens and necessitating alternative antigen-targeting strategies. TAAs remain an important class of vaccine targets across tumor types. MUC1 and CAIX are highly expressed in ccRCC, whereas 5T4 is prevalent in pRCC, with reported expression in ∼71%-84% of ccRCC tumors and ∼92% of pRCC tumors, respectively.^55^^,^^57^^,^^64^ Although MUC1 is also expressed in a high proportion of chRCC tumors (∼91%), its predominantly cytoplasmic localization may restrict antibody-mediated targeting. However, vaccine strategies targeting HLA-restricted MUC1 peptides remain a viable strategy for eliciting T cell responses.^55^

Beyond classical TAAs, hERVs have emerged as a promising antigen source.^80^ In ccRCC, a set of hERVs are regulated by HIF-2α and some hERV-derived peptides can elicit CD8^+^ T-cell responses.^81^^,^^82^ Although hERVs continue to be explored as potential antigen targets in ccRCC, their landscape in pRCC and chRCC remains largely unexplored, representing a potential reservoir of shared ‘public’ antigens.^83^

Importantly, the effectiveness of any vaccine strategy depends not only on antigen selection but also on intact antigen presentation.^84^ Although complete HLA class I loss-of-function mutations are rare in RCC, coordinated downregulation of APM components such as TAP1 and LMP2 occurs in ∼40% of ccRCC and pRCC tumors and up to 80% of chRCC tumors.^85^ Likewise, two independent IHC studies of paraffin-embedded ccRCC samples reported reduced HLA-I expression in ∼38% of cases, each linking low expression with significantly worse prognosis.^86^^,^^87^ In chRCC, broad suppression of HLA-A/B/C and multiple APM components results in defective peptide loading and a profoundly ‘immune-cold’ phenotype, imposing a potential barrier to effective T cell-based vaccination strategies.^88^

#### Tumor-intrinsic immune suppression: barriers to vaccine efficacy

The RCC tumor immune microenvironment (TIME) is highly heterogeneous, with each subtype—and even molecular subtypes within a histologic subtype—exhibiting distinct patterns of immune infiltration and responses to various therapeutics.^89^ In ccRCC, in most advanced disease stages, there is an accumulation of terminally exhausted CD8+ T cells and reduced T cell receptor diversity.^90^ In contrast, chRCC exhibits a profoundly immune-desert TIME, with low overall immune cell infiltration, and CD8^+^ T cells constituting only a small fraction of CD45+ T cells that are in the tumor. Among the few CD8+ T cells that are present, they demonstrate minimal inhibitory checkpoint expression [e.g. Programmed cell death protein 1 (PD-1)], limited clonal expansion, and poor tumor specificity, suggesting bystander rather than tumor-specific populations.^88^

Although the TIME of pRCC is still incompletely characterized, bulk transcriptomic analysis and IHC studies have revealed distinct biological subtypes, an ‘immune-enriched’ and ‘immune-low’ group. The immune-enriched phenotype is paradoxically associated with poorer OS, similar to what has historically been observed in ccRCC.^91^ Overall, T cell infiltration in pRCC is lower the ccRCC but higher than chRCC.^92^ Whether through exhaustion or absence, CD8^+^ T cell dysfunction is a defining feature of RCC subtypes and directly influences how each tumor type responds to immunotherapy.

Beyond the T cell compartment, macrophages are among the most abundant and immunologically impactful cell populations in RCC. Tumor-associated macrophages (TAMs) exert potent immunosuppressive effects on CD8^+^ T cells, thereby promoting and sustaining a terminally exhausted phenotype.^93^, ^94^, ^95^ In ccRCC, there is an observed shift from proinflammatory to immunosuppressive macrophages as disease advances from early to late stages, with TAMs expressing ligands for numerous T cell immune checkpoints including Programmed death-ligand 1 (binds to PD-1), CD80/CD86 (binds to CTLA4), Nectin-2 and PVR (binds to TIGIT), Galectin-9 (binds to TIM-3), HVEM (binds to BTLA), and SPP1 (binds to CD44).^90^ Although chRCC has a lower overall immune infiltration, there is a higher relative proportion of myeloid cells compared with ccRCC, with TREM2+ TAMs expressing high levels of TIM-3 and FGL2.^88^^,^^96^ Emerging data for FGL2 suggest that FGL2 on TAMs interacts with FcγRIIB+CD8+ T cells to induce apoptosis in human and mouse melanoma models, providing at least one potential reason for low intratumoral T-cell density in chRCC.^97^ Multiplex immunofluorescence and spatial transcriptomics comparing immune cell architecture between pRCC and ccRCC show that pRCC has higher cell clustering of immunosuppressive macrophages.^98^ Higher presence of these adverse TAM populations may be linked to pRCC tumors expressing high levels of ABCC2, which can shape the TME in a manner that dampens effective antitumor immune responses.^99^ Given these inhibitory myeloid programs, overcoming TAM-mediated suppression remains essential for improving the efficacy of T cell-based vaccine strategies in RCC.

#### Effector reinvigoration: ICI and rational combinations

Several vaccine trials discussed thus far have attempted to counter the immunosuppressive TME in RCC, such as combining vaccines with sunitinib in ADAPT and IMPRINT or using cyclophosphamide pretreatment in IMPRINT. However, these strategies were insufficient to improve clinical outcomes, underscoring the need for improved rational combinations that directly target dominant immunosuppressive mechanisms. Combining ICIs with vaccination may provide greater clinical benefit, as ICIs have shown meaningful efficacy across cancers, particularly in metastatic ccRCC.^100^, ^101^, ^102^ Because ccRCC frequently harbors exhausted CD8^+^ T cells expressing high levels of PD-1, TIM-3, LAG-3, and TIGIT, immunotherapies targeting these inhibitory pathways may synergize with vaccines by protecting newly primed T cells from suppression and enhancing their antitumor activity.^90^ Further, as combinations of ICI plus TKI are frequently utilized in RCC, it remains to be seen whether this would also be an effective cotherapy with therapeutic vaccines. There is literature to support the concept that specific TKIs, such as cabozantinib, may reduce suppressive myeloid populations, and therefore have more pronounced benefit in the setting of a high myeloid infiltration in the tumor.^103^ Conversely, although initial preclinical models argued for ‘regularization’ of a dysfunctional vasculature by TKIs (potentially improving T cell infiltration), emerging clinical data suggest that TKIs are more likely to lead to ‘vascular pruning’, with decreased microvascular density in the tumor.^104^ Of note, the phase III IMPRINT trial of a TAA vaccine in combination with sunitinib suggested possible worse outcomes than with sunitinib alone,^66^ suggesting potential caution when combining vaccines with TKIs. Overall though, the true impact of combining therapeutic cancer vaccines with IO+TKI remains largely undefined and is in need of further investigation.

Unlike ccRCC, IO-based combination trials in pRCC and chRCC including KEYNOTE-B61 and SUNIFORECAST have shown more modest efficacy.^105^^,^^106^ Consistent with these clinical outcomes, pRCC and chRCC exhibit reduced expression of immune checkpoint receptors targeted by contemporary ICIs, including PD-1 and CTLA-4, alongside evidence of diminished antitumor T cell activity.^107^ Attempts to explore a combination of mTOR and TKI inhibitors are few in number and underpowered, preventing meaningful conclusions.^108^ Together, these findings may suggest more limited benefit from pairing current ICIs with a vaccine in pRCC and chRCC.

Emerging biologic targets that could synergize with a chRCC-directed vaccine include components of the ferroptosis pathway.^109^ ChRCC tumors exhibit markedly elevated glutathione levels driven by SLC7A11 and GPX4 upregulation, which protects them from ferroptosis,^110^ and studies have demonstrated that inhibiting SLC7A11 or FSP1 effectively triggers ferroptotic death in chRCC models.^111^^,^^112^ Notably, ferroptosis induction has been shown to upregulate MHC-I, activate IFN-γ/STAT1 signaling, drive macrophage polarization toward an M1-like phenotype, promote dendritic-cell maturation, enhance CD8+ T cell infiltration, and potentiate ICI responses—collectively suggesting that ferroptosis-targeting strategies could convert cold chRCC tumors into more inflamed, vaccine-responsive cancers.^113^, ^114^, ^115^ Future studies are required to determine whether these potential immunologic effects could translate to chRCC, as direct validation in preclinical or clinical settings is still needed.

## Future direction

Therapeutic cancer vaccines in RCC have evolved from broad autologous whole tumor approaches to more precise, personalized neoantigen-targeting strategies, in large part enabled by the development of next-generation sequencing and advanced computational tools. Vaccine design now spans fully personalized neoantigen vaccines, partially off-the-shelf multi-epitope constructs, and fully off-the-shelf TAA platforms, enabling both precision and broader accessibility. Advances in sequencing, computational pipelines, and antigen discovery allow these approaches to target patient-specific mutations, shared tumor antigens, or a combination thereof, tailoring immune activation to tumor biology. As the field continues to expand the known antigen landscape of RCC tumors, there will be more potential therapeutic targets for vaccine-based approaches. Selection of proper antigens, ideally ones that are tumor-specific, will be needed to translate into clinical activity.

**Figure 2:**
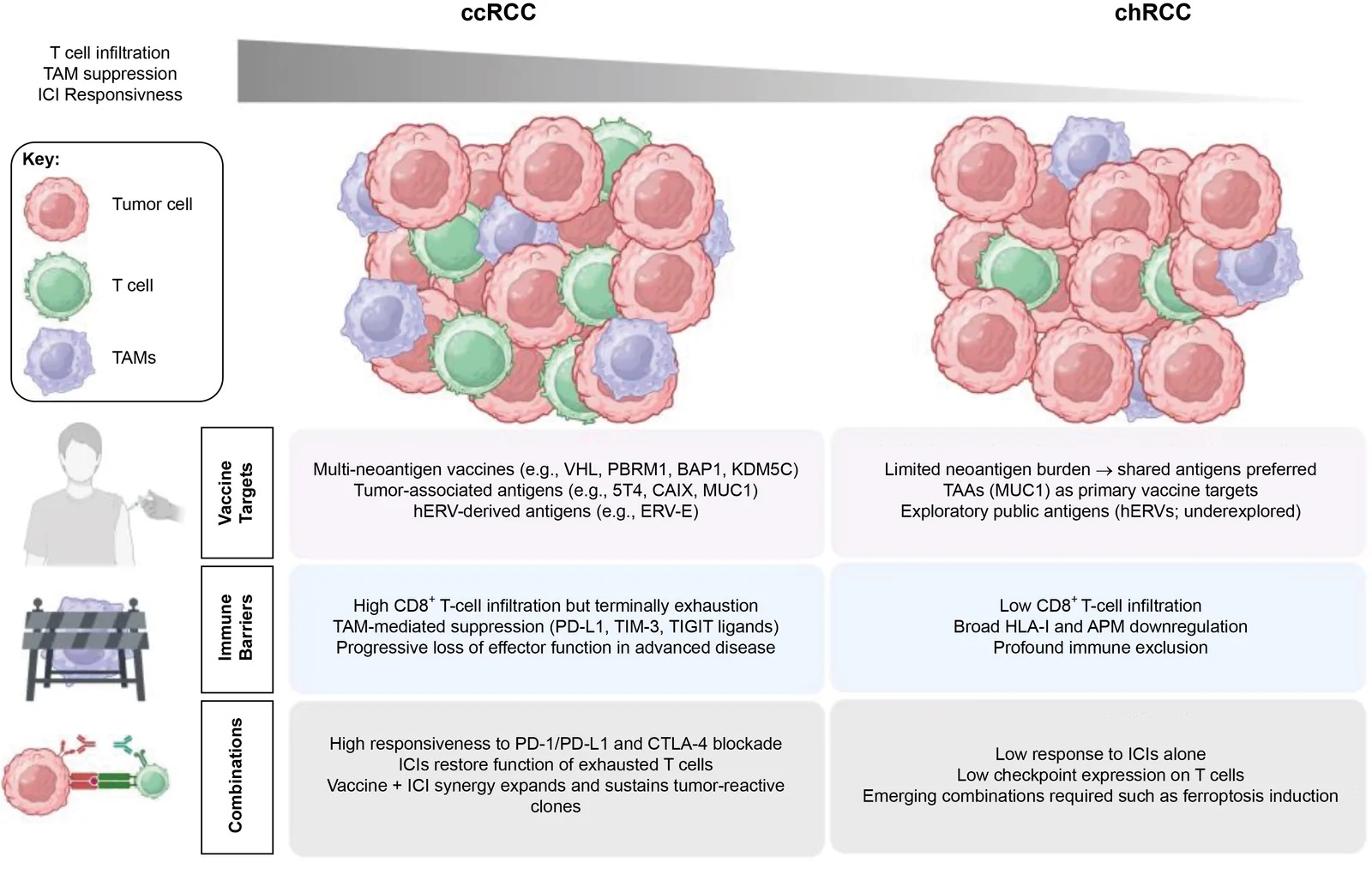
**RCC subtype-specific considerations for cancer vaccine design and combination strategies.** Schematic comparison of vaccine targets, immune barriers, and therapeutic combinations for vaccination in ccRCC versus chRCC. ccRCC is characterized by higher CD8+ T cell infiltration with concurrent TAM-mediated suppression and T cell exhaustion, supporting strategies that combine multi-antigen vaccines with ICIs to restore effector function. In contrast, chRCC displays low T cell infiltration, broad HLA/APM downregulation, and immune exclusion, favoring approaches that prioritize limited neoantigen targets and therapies that enhance antigen presentation and T cell recruitment. Together, these subtype-specific immune contexts highlight the need for tailored vaccine strategies and rational combination therapies across RCC subtype. APM, antigen-processing machinery; CAIX, carbonic anhydrase IX; CcRCC, clear cell renal cell carcinoma; chRCC, chromophobe renal cell carcinoma; hERV, human endogenous retroviruses; HLA-1, human leukocyte antigen; ICIs, immune checkpoint inhibitors; MUC1, mucin 1; PD-1, Programmed cell death protein 1; PD-L1, Programmed death-ligand 1; RCC, Renal cell carcinoma; TAAs, tumor-associated antigens; TAM, tumor-associated macrophage; VHL, von Hippel–Lindau.

Further, the clinical setting is key. Early RCC vaccine trials support the concept that vaccination alone may have difficulty clearing ‘bulky’ macrometastatic disease. This may be due to a number of factors, including inability to generate enough tumor-specific T cells, and the need to overcome an increasingly immunosuppressive TME in advanced disease. Currently, the minimal disease setting such as adjuvant after resection or treatment of oligometastatic disease is an appealing clinical setting for therapeutic vaccination. To further improve efficacy, particularly in the metastatic setting, effective cotherapies will be required. Further, RCC subtype-specific considerations are critical—for example, ccRCC often harbors exhausted but infiltrated T cells amenable to combination ICI-vaccine approaches, whereas chRCC exhibits an immune-desert phenotype, requiring novel interventions to improve T cell infiltration, such as ferroptosis induction to enhance antigen presentation and T cell recruitment (Figure 2). Together, these insights emphasize that reviving cancer vaccines in RCC will require stage- and subtype-informed strategies, integrating antigen breadth, personalized targeting, and TME modulation across adjuvant and metastatic settings.
